# Treatment of Interstitial Gingivitis

**Published:** 1909-03-15

**Authors:** Eugene S. Talbot

**Affiliations:** Chicago


### TREATMENT OF INTERSTITIAL GINGIVITIS.

BY EUGENE S. TALBOT, M.S., D.D.S., M.D., L.L.D., CHICAGO.

Fifteen years' continuous study of inflammation of the gums and pericementum and absorption of the alveolar process have resulted in a line of rational treatment based on scientific methods. Researches have shown that, in both animals and man, evolution—due to environment and disease under the law of economy of growth, or use and disuse of structures—is the foundation. If the law of evolution could be reversed and our progeny educated to return to primitive methods of using the jaws in mastication, this disease, as well as deformities of the jaws, irregularities of the teeth, and tooth-decay, would be far less prevalent. To accomplish this would require special training and selection. Generations of persistent effort would be necessary to carry out this reform before the ideal race could be produced. In the meantime we must meet these conditions as we find them, and how best to accomplish this demands the attention of investigators.

It has been demonstrated many times that interstitial gingivitis is due to both constitutional and local causes. In the treatment of constitutional interstitial gingivitis, it must be borne in mind that the patient always has a tendency to other grave disorders, the symptoms of which can be easily determined and which, if allowed to remain, will eventually produce disastrous results.

The accumulation of waste products not properly eliminated and circulating in the blood-stream interferes materially with metabolism, and self-poisoning or auto-intoxication is the inevitable result.

These various poisonous substances have in some cases been accumulating in the blood and tissues for years, and it becomes very necessary not only to demonstrate their presence, but in some way to estimate the amount of poisonous material in the blood, so that we may have some guide as to the persistence required in administering the proper method of treatment. At present, the only method of locating these poisons is the examination of the urine, and the definite indications are excessive urinary acidity and indicanuria.

When a patient presents himself suffering from interstitial gingivitis, first inquire carefully into his history; viz, his family—married or single; habits—sedentary or otherwise; diet—use of stimulating foods and drinks; hereditary factors—if any; general health—as to pain in joints, muscles, headache, rheumatism, asthma, constipation, kidney, liver or venereal disorders, tuberculosis, stomach disorders, etc. An intelligent digestion of the data elicited will give a definite idea as to the line of treatment. Have the patient save the urine voided during twenty-four hours—this must be measured to ascertain the quantity passed in twenty-four hours. Examine this for the degree of acidity and indican only, if the patient is under thirty years of age. The average dentist can easily do this. If the patient is above thirty it is advisable to have a thorough urinary examination made by some medical laboratory to ascertain in addition if albumin, sugar, pus, blood, or casts are present. If such conditions are present, the patient should be informed and recommended to a physician. If the urinary acidity is high—above forty degrees—and indican is present, they should be gradually reduced to the normal (30 to 40 degrees) by the use of suitable alkaline remedies and intestinal antiseptics..

During the past twelve years I have done considerable experimenting along these lines, and through the kindness of the Abbott Alkaloidal Company I have been able to have my preparations compounded. The following formula, after some minor changes, has finally been adopted, having given constant satisfaction. This combination has proved to be of such value that I am now giving it to the profession. Each teaspoonful (60 grains) contains:

| | | |
|---|---|---|
| Sodiom bicarb., | 40 | grains |
| Sodium sulfate, | 10 | " |
| Sodium sulfocarb., | 5 | " |
| Colchicin, | 1-250 | grain. |
| Juglandin, | 1-3 | " |
| Xanthoxylin, | 1-3 | " |
| Aromatics, q. s. | | |

One-half to one teaspoonful should be given in a glass of hot or warm water one-half to one hour before meals. This preparation will rapidly diminish a high urinary acidity and reduce a urine abundant in indican to the normal. If the urinary acidity is below thirty degrees, and less than forty-eight ounces of urine are passed in twenty-four hours, the patient should increase the amount of water taken in twenty-four hours. The normal amount of liquids in twenty-four hours should equal ten to twenty glasses of pure water. A second urinalysis for acidity and indican after two or three weeks will indicate the result of the treatment.

The orthodox local treatment of removing all irritation must be thorough. Pyorrhea alveolaris (pus infection) cannot be present without there first being an interstitial gingivitis. Remove the interstitial gingivitis, and the pyorrhea alveolaris will take care of itself. To reduce the inflammation and to restore the gums and the alveolar process to a normal condition requires more than the mere application of drugs to the surface of the gums. The inflammation, being deep-seated, requires the application of some drug that will penetrate into the alveolar process as well as the gum tissue, and reach the arterial system in the bone. No preparation will accomplish this as well as iodin. To further improve the power of absorption, glycerine is an excellent medium, and to further increase its usefulness, zinc iodid has been added. The following prescription applied locally upon the gums twice per week will be of great benefit.

*Iodo-glycerole.* (Talbot.)

| | | |
|---|---|---|
| Zinc iodid, | 15 | grains. |
| Water, | 10 | " |
| Iodin, | 25 | " |
| Glycerin, | 50 | " |

This will make nearly two fluid drams. The use of a tooth-powder or paste to which has been added a small amount of C. P. zinc sulfocarbolate rubbed into the gums with the gum-massage brush is of value. Severe cases seem to progress more rapidly when the mouth is rinsed four or five times a day with fifteen per cent. zinc sulfocarbolate.

Since disuse of the structures is a factor in this disease, constant friction is necessary, and a gum-massage brush—not a tooth-brush—should be used to stimulate the gums.

In 1886 I commenced a systematic study of brushes for gum-massage purposes.

The bristles of the brush should be unbleached and should be made of two grades, medium and hard. Two brushes should be used, one every other day, to allow the bristles to become thoroughly dry. A soft brush is of no value and should never be used, since the desired results cannot be obtained. A gum wash, to be used with the gum massage for the purpose of stimulating and contracting the gums and also for destroying the bacteria in the mouth, is composed of the following:

| | | |
|---|---|---|
| Zinc sulfocarb., | 5 | per cent |
| Alcohol, | 30 | " |
| Water, | 65 | " |
| Oil wintergreen, q. s. | | |

I have obtained good results in those cases of long standing where the disease has been allowed to progress until the teeth have slightly loosened, especially if the patients are young, by placing them on whole wheat or rye bread, requiring them to masticate thoroughly, thus in a measure restoring the law of use where disuse of structures formerly prevailed. While the treatment is proceeding the patient's diet and habits must be properly regulated so as to prevent the further formation of an excess of acids and indican in the system.—Feb. 1909 *Cosmos.*

